# Dynamic Tensile Properties and Energy Dissipation of High-Strength Concrete after Exposure to Elevated Temperatures

**DOI:** 10.3390/ma13235313

**Published:** 2020-11-24

**Authors:** Nao Lv, Hai-bo Wang, Qi Zong, Meng-xiang Wang, Bing Cheng

**Affiliations:** 1 State Key Laboratory of Mining Response and Disaster Prevention and Control in Deep Mine, Anhui University of Science &Technology, Huainan 232001, China; lvnao1990@163.com (N.L.); qzong@aust.edu.cn (Q.Z.); mxwang@aust.edu.cn (M.-x.W.); dzcx1995@163.com (B.C.); 2 School of Civil Engineering and Architecture, Anhui University of Science &Technology, Huainan 232001, China

**Keywords:** high-strength concrete, elevated temperature, splitting tensile strength, fracture characteristic, energy characteristic

## Abstract

In view of the devastating outcomes of fires and explosions, it is imperative to research the dynamic responses of concrete structures at high temperatures. For this purpose, the effects of the strain rate and high temperatures on the dynamic tension behavior and energy characteristics of high-strength concrete were investigated in this paper. Dynamic tests were conducted on high-strength concrete after exposure to the temperatures of 200, 400, and 600 °C by utilizing a 74 mm diameter split Hopkinson pressure bar (SHPB) apparatus. We found that the quasi-static and dynamic tensile strength of high-strength concrete gradually decreased and that the damage degree rose sharply with the rise of temperature. The dynamic tensile strength and specific energy absorption of high-strength concrete had a significant strain rate effect. The crack propagation law gradually changed from directly passing through the coarse aggregate to extending along the bonding surface between the coarse aggregate and the mortar matrix with the elevation of temperature. When designing the material ratio, materials with high-temperature resistance and high tensile strength should be added to strengthen the bond between the mortar and the aggregate and to change the failure mode of the structure to resist the softening effect of temperature.

## 1. Introduction

With the rapid development of high-rise buildings and long-span buildings, the requirements of concrete strength and mechanical properties have gradually increased. High-strength concrete is usually prepared by adding silica fume and fly ash, using small aggregate, and reducing the water:cement ratio [1]. The mixing, pouring, and curing of high-strength concrete is similar to that for conventional concrete, which makes it possible for the material to be applied on a large scale [2]. As a result, high-strength concrete is increasingly used in buildings and structures where performance is required within a limited space/volume [3].

With the rapid development of urbanization and the high density of high-rise buildings, the frequency of building fires has increased in recent years. Chemical explosions, gas explosions, and man-made explosions from terrorist attacks will lead some buildings to face fire and impact or explosion loads [4]. When concrete is subjected to fire, the residual mechanical properties can be seriously reduced by thermal damage due to internal physical and chemical changes, cracks, and deformations [5,6,7]. Although it has higher mechanical properties when compared with normal-strength concrete, high-strength concrete can be more vulnerable to high temperatures. Therefore, the high-temperature mechanical properties of high-strength concrete have aroused the interest of researchers.

The compressive strength is considered as the representative of the mechanical properties of concrete. Li et al. [8], Cheng et al. [9], and Ghandehari et al. [10] conducted a series of tests to study the behavior of high-strength concrete under elevated temperature. They found that there was an unavoidable reduction for the compressive strength of concrete when exposed to high temperatures. From room temperature to 300 °C, the compressive strength of concrete stayed constant or even increased slightly. Then, from 300 °C to 800 °C, the compressive strength of concrete decreased dramatically. Almost all the compressive strength of concrete was lost under high temperatures above 800 °C.

Concrete has a strain rate effect, and the split Hopkinson pressure bar (SHPB) test and drop hammer test are typically applied to study the dynamic mechanical performance of concrete [11,12,13]. The ranges of the strain rate of SHPB and drop hammer are 10–10^3^ s^−1^ and 10^−1^–10 s^−1^, respectively [14]. Among them, the SHPB device is widely used to study the impact performance of materials under medium and high strain rates due to its simple structure and easy operation. The dynamic compressive properties of concrete under high temperatures have been studied extensively. From these studies, the residual compressive strength was shown to decrease as the temperature rises and to increase approximately linearly with the increase of the strain rate logarithm [15,16,17].

The damage of concrete structures in engineering is often affected by the tensile properties. In the process of uniaxial compression, the damage of concrete is essentially controlled by the tensile damage perpendicular to the direction of the pressure action [18]. Under the action of an earthquake, concrete structures often suffer from oblique tension failure and shear tension failure. This occurs suddenly without any warning and typically leads to disastrous consequences.

The tensile strength plays a vital role in determining the tensile, shear, and impact resistance of columns, beams, and other components. Particularly at high temperatures, the degradation of tensile strength should be carefully evaluated given that tensile strength could be more severely damaged than compressive strength [19,20]. Therefore, it is of great engineering and scientific significance to study the dynamic splitting tensile properties of high-strength concrete after elevated temperatures.

Researchers suggested that the splitting tensile strength of conventional concretes decreased to almost zero under high temperatures over 600 °C [19]. Wasim Khaliq [21,22] proposed that a temperature between 300 and 400 °C may be a critical temperature beyond which the tensile strength will be lost much faster than at lower temperatures. The rapid decrease in the splitting tensile strength over 400 °C could be due to excessive cracks caused by thermal stress and component incompatibility [21,23,24].

Jin [25] conducted experimental research on the splitting tensile strength of C40 (the standard compressive strength is 40 MPa) high-performance concrete cube specimens, analyzed and discussed the mechanism of the concrete material degradation after different high temperatures and the influence on the splitting tensile strength, and discussed the law of the splitting tensile strength changing with the temperature.

Zhou et al. [26], Qin et al. [27], Shi et al. [28], and Guo et al. [2] analyzed the dynamic tensile properties of high-strength concrete at room temperature through a series of dynamic splitting tensile tests and numerical simulations. They found that the dynamic fracture tensile failure behavior of concrete had the strain rate effect and dynamic compression and showed different fracture failure modes with those in quasi-static states. Previous investigations rarely studied the dynamic splitting tensile behavior of concrete after high temperatures.

Jin [29] considered the internal heterogeneity of concrete and integrated the degradation of the mechanical properties and strain rate effect of dynamic loading. The high-temperature splitting and pulling behavior of concrete were analyzed by mesoscale simulation. However, the use of round aggregate particles in the simulation is quite different from the actual aggregate shape, and this cannot fully demonstrate the tensile properties of concrete at high temperatures.

Liang [30] studied the coupled effects of temperature and impact loading on the tensile strength of an ultra-high performance fiber reinforced concrete (UHPFRC) and made a comparison between the tensile strength and compressive strength of UHPFRC obtained in the hot state and cooled-down state. However, the specimen did not contain coarse aggregate and, thus, cannot reflect the failure mode of concrete containing coarse aggregate at high temperatures.

In the current work, after a brief introduction of the whole experimental process, our test results on the basic static mechanical properties (compressive strength at room temperature and splitting tensile strength after elevated temperatures) of high-strength concrete are reported. The dynamic behaviors of high-strength concrete after elevated temperatures were described by the SHPB experiment, namely, the fracture morphology, stress–strain relation, dynamic splitting tension strength, and energy absorption ability. We analyzed the effects of elevated temperatures and the strain rate on the mechanical performances of high-strength concrete. The results provide a reference for evaluating the dynamic splitting tension performance of concrete after high temperatures and the fire resistance.

## 2. Experimental Scheme and Theoretical Basis 

### 2.1. Materials and Preparation of Specimens

Silica fume and fly ash could improve the refinement of the pore structure, strengthen the interface transition zone (ITZ), improve the workability, and enhance the bonding effect, thereby, significantly increasing the strength of the concrete [31,32]. Based on the mixing proportion of C60 (the standard compressive strength is 60 MPa) strength concrete in [33], silica fume and fly ash were added, and the mixing proportion was adjusted slightly. The mixture proportions of high-strength concrete are listed in Table 1.

Although high-strength concrete usually possesses better mechanical properties than normal-strength concrete, it may easily burst after exposure to elevated temperatures. To achieve the fire resistance and high-temperature crack resistance of concrete, a small amount of rubber powder was added [34]. The cement was ordinary Portland cement (P.O42.5), the silica fume was produced in a factory in Sichuan Province, and the composition of the cement and silica fume are shown in Table 2.

River sand with a density of 2600 kg/m^3^, a maximum particle size of 5 mm, and a specific gravity of 2.70 was used as fine aggregate. The coarse aggregate was a crushed stone with a particle size of 5–15 mm. The water used in the test was tap water obtained from the laboratory. The water-reducer was polycarboxylic acid superplasticizer (Type F) [35]. The fly ash was 1250 mesh first-grade fly ash. The test used waste tire rubber powders with a particle size of 40 mesh.

The International Society for Rock Mechanics recommends that the specimen thickness used in the Brazilian splitting tensile test should be approximately equal to half of the diameter; that is, the ratio of thickness to diameter is about 0.5 [36]. As a brittle material, concrete can use to the rock standard for size selection in the splitting tensile test [2]. In this test, the dimensions of dynamic splitting specimens were 70 mm in diameter and 35 mm in thickness. To form a sharp contrast with the dynamic test, the static tension test used the same size specimens as the dynamic splitting test. As the maximum particle size of the aggregate used in this test was 15 mm, which is less than 31.5 mm, the size of the static compression specimens was 100 mm × 100 mm × 100 mm [37]. Compared with the standard size, the measured compressive strength should be multiplied by a factor of 0.95 [37]. 

The specimens were prepared using the pouring method, and the controlled non-parallelism of the specimens end faced as far as possible to prevent the specimens from being damaged in advance due to uneven loading. The specimens were removed after 24 h. According to the ASTM C192 standard [38], the specimens were tested for high-temperature heating using a SX-5-12 box-type resistance furnace after 28 days of standard curing. The resistance furnace temperature rise rate was 6 °C /min, and the highest temperatures could reach up to 1200 °C. Each group of specimens was heated to the corresponding temperature and kept constant for 2 h. They were cooled naturally in the furnace to room temperature, and all specimens showed no high-temperature bursting.

### 2.2. SHPB Test Equipment and Basic Principle

**Figure 1:**
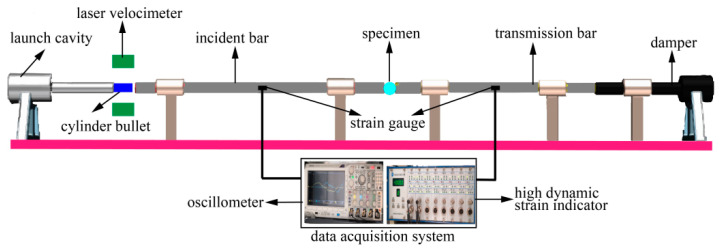
Schematic diagram of the split Hopkinson pressure bar (SHPB) system.

The dynamic splitting tensile test of concrete specimens was conducted on the 74 mm SHPB test system. The striker bar length was 0.40 m, and the lengths of the incident bar and transmission bar were 3.20 m and 1.80 m, respectively. The striker bar, incident bar, and transmission bar of the SHPB device were alloy steel, with a density of 7.8 g/cm^3^, an elastic modulus of 210 GPa, and a longitudinal wave velocity of 5190 m/s. The experimental device is shown in Figure 1.

The experimental method is to measure the distribution of the incident waves, reflected waves, and transmitted waves in the bar with the super dynamic strain indicator and then to calculate and analyze the stress–strain relationship according to the propagation theory of the one-dimensional elastic stress wave. In the incident bar, the strain gauges were all resistance strain gauges with a model of BX120-3AA and a sensitivity coefficient of 2.08 ± 0.01. In the dynamic splitting experiments, to accurately collect the weak transmission pulse, semiconductor strain gauges with a resistance of 120 Ω and a sensitivity coefficient of 110 ± 0.05 were adopted in the transmission bar.

Elasticity analysis [39,40] shows that the diametral compression generates a uniform transverse tension along the vertical loading plane as can be determined by Equation (1).

(1) $$f_{t}=\frac{2P}{\pi DL}$$

where *P* is the compressive force applied to the specimen, and *D* and *L* are the specimen diameter and length, respectively. The tensile stress generated ultimately splits the specimen into two halves along the vertical diameter, and the tensile strength can be derived from the maximum force sustained by the specimen using Equation (1).

Gomez [41] used photoelasticity to derive the specimen stress distribution in dynamic splitting tests and proposed that when the specimen experiences stress equilibrium, the dynamic stress distribution is the same as that under static loading, and Equation (1) can be used to estimate the dynamic tensile stress at the center of the specimen. However, both the elastic analysis and the photoelastic experiment were based on homogeneous materials. In contrast, concrete is uneven on a mesoscale. Guo [2] determined through elastic-plastic analysis and experimental measurements that static elastic analysis could also be used to estimate the stress distribution of dynamic split specimens for heterogeneous concrete once the stress balance was reached. In the SHPB dynamic splitting tensile test, the experimental data can be processed as follows, Equations (2)–(4):

(2) $$\dot{\varepsilon }(t)=\frac{C_{0}}{D}[\varepsilon_{i}(t)-\varepsilon_{r}(t)-\varepsilon_{t}(t)]$$

(3) $$\varepsilon (t)=\frac{C_{0}}{D}\int_{0}^{t}\left[\varepsilon_{i}(t)-\varepsilon_{r}(t)-\varepsilon_{t}(t)\right]\mathrm{dt}$$

(4) $$\sigma_{t}(t)=\frac{2AE}{\pi DL}\varepsilon_{t}(t)$$

where *έ*(*t*), *ε*(*t*) and *σ_t_*(*t*) are the strain rate, strain, and stress of the specimen; *A* is the cross section area of the compression bar; *E* and *C*_0_ are the compression bar material elastic modulus and wave velocity, respectively; *ε_i_*(*t*), *ε_r_*(*t*), and *ε_t_*(*t*) are the incident strain, reflected strain, and transmitted strain of a certain time *t*, respectively; and *t* is the duration of the stress wave.

## 3. Test Results and Analysis

### 3.1. Static Test

**Figure 2:**
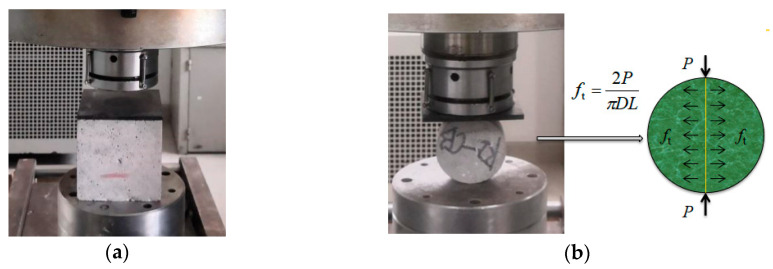
Loading modes of (**a**) static compressive and (**b**) splitting tensile tests.

At present, the direct tensile test and splitting tensile test were generally adopted to the static and dynamic tensile strength of brittle materials, such as concrete and rock [42,43]. As it is difficult to make the axis of tensile load coincide with the specimen axis in the direct tensile test, the experimental data of the direct tensile test is usually inaccurate [1]. The splitting tensile test is generally used for the tensile strength test to make up for the defects of the direct tensile test. The static compressive and splitting tensile tests were carried out using the rock mechanics testing machine as the corresponding strain rate was relatively low, as shown in Figure 2. The static compressive strength was 60.08 MPa at room temperature.

For splitting tensile tests of this nature, steel strips are typically placed between the specimen and the loading platens to help distribute the load onto the specimen. In this test, such steel strips were not used, mainly because the addition of the steel strips made it difficult to place the specimen when the SHPB device was used for the dynamic splitting test. In addition, the reflection of the waves existed at the interface between the steel strips and the specimen, which disturbed the incident wave, reflected wave, and transmitted wave measured by the strain gauge resulting in complicated and inaccurate data processing.

The steel strips were also not used in the static splitting test to compare the dependence of the quasi-static and dynamic tensile values on the strain rate. Compared to results derived from tests where steel strips were used, the tensile strengths were typically 8% lower in splitting tests without the incorporation of steel strips [2]. Taking the average of the test results, the static tensile strengths after room temperature, 200, 400, and 600 °C were 3.35, 3.04, 1.79, and 1.07 MPa, respectively.

**Figure 3:**
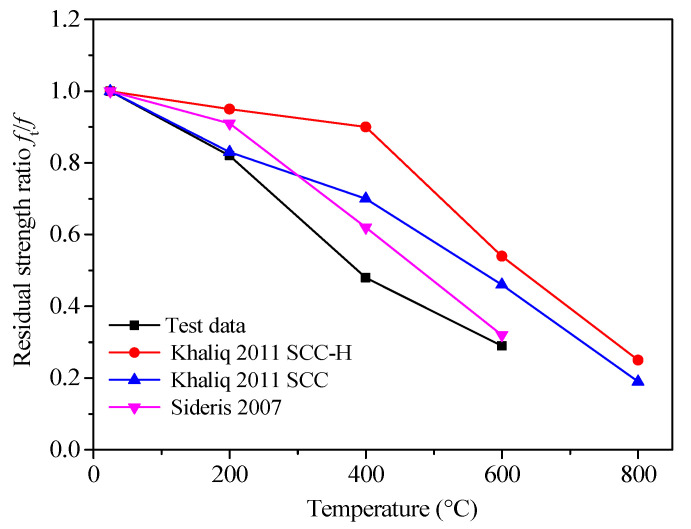
The relationships between the residual tensile strength factor and temperature.

The ratio of the splitting tensile strength at specified temperatures to the splitting tensile strength at room temperature (*f*_t_/*f*) is the residual tensile strength factor. Combined with the data of this experiment and those compiled from different references [21,44], the relationships between the residual tensile strength factor and the temperature of concrete are plotted in Figure 3.

As shown in Figure 3, the residual tensile strength factors of the concretes from the test and references [21,44] showed variation that can be attributed to investigation factors, such as different test methods, mix proportions, test techniques, test conditions, and heating regimes adopted by different researchers [44,45]. However, with the rise of temperature, the residual tensile strength factors tended to decrease.

### 3.2. Dynamic Splitting Tensile Test

The dynamic splitting tension tests of high-strength concrete specimens after the high temperatures of 200, 400, and 600 °C were conducted using a SHPB device with four kinds of impact pressure (0.15, 0.20, 0.25, and 0.30 MPa). Simultaneously, for the convenience of analysis and comparison, the dynamic splitting tensile test of concrete specimens at room temperature (25 °C) was conducted. In the dynamic loading process, the compression strain rate at each moment was obtained using Equation (2), and the average value of the strain rate platform segment was taken as the loading strain rate. Experimental results are shown in Table 3, and the results interpretation will be in the following chapters.

The damage degree *D* and the dynamic strength growth factor *DIF* (the ratio of dynamic strength to static strength) of concrete after high temperatures can be calculated using Equations (5) and (6), respectively.

(5) $$D=1-{\left(\frac{c_{2}}{c_{1}}\right)}^{2}$$

(6) $$DIF=\frac{f_{\mathrm{td}}}{f_{\mathrm{ts}}}$$

where *c*_1_ is the wave velocity at room temperature of the specimen; *c*_2_ is the wave velocity after a specific temperature treatment of specimen; and *f*_td_ and *f*_ts_ are the dynamic and static tensile strength, respectively.

#### 3.2.1. Analysis on Typical Waveform Curve of SHPB 

**Figure 4:**
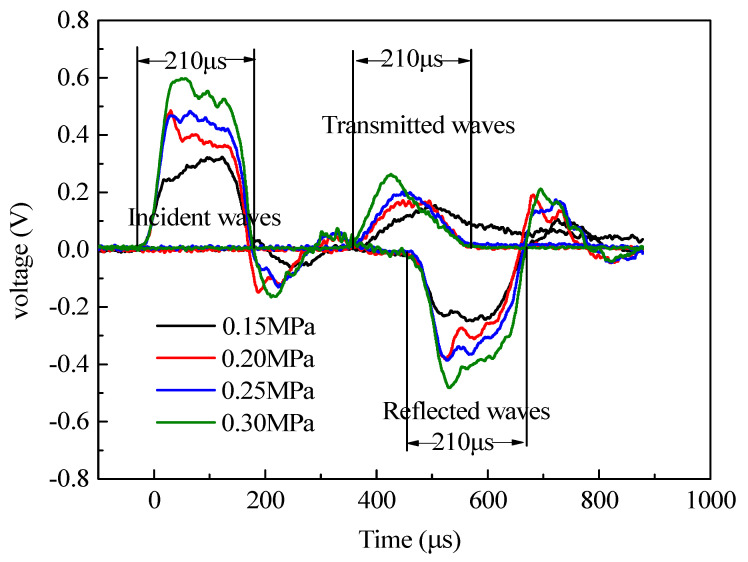
Typical voltage time-history curves.

The typical SHPB voltage time-history curve was obtained according to the experimental data collection as shown in Figure 4.

From Figure 4, the action time of the incident waves, reflected waves, and transmitted waves were all 210 μs, indicating that the action time of the stress waves was relatively stable. The reflected wave was during the second half to the platform, which showed that the strain rate of the specimen closed to a constant, and that the system could realize constant strain rate loading. The increase in the stress wave amplitude in the incident bar led to the gradual increase in the stress wave amplitude in the transmission bar. With the rise of the impact pressure, the transmitted stress corresponding to the same time increased, and with the shorter time, the transmitted wave reached the peak stress.

**Figure 5:**
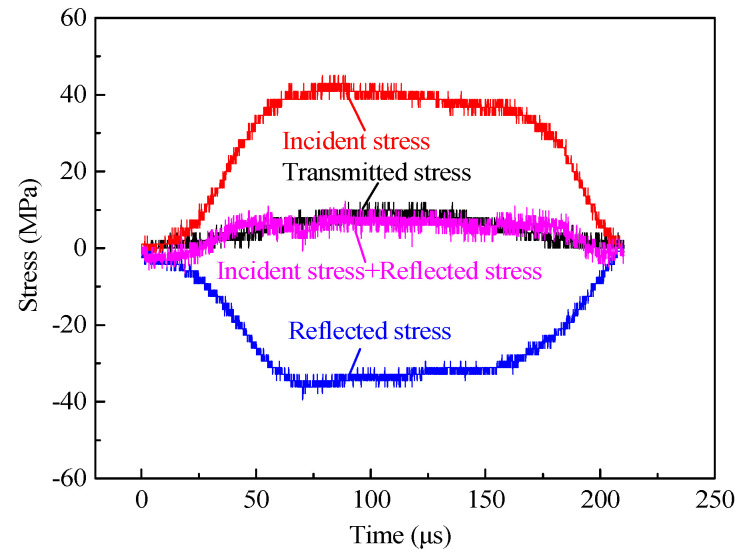
Stress-balance check of the split Hopkinson pressure bar (SHPB) dynamic splitting tensile tests.

From Section 2.2, the experimental data was processed based on the stress balance. To ensure the real reliability of the experimental data, the stress balance in the experimental process must be verified. According to the one-dimensional stress wave principle and stress uniformity hypothesis, the stress at the incident end should be the same as that at the transmitter end. If the superposition results of the incident wave and the reflected wave were equal to that of the transmitted wave, this indicates that the specimen was in the stress balance state. The stress balance verification is shown in Figure 5.

The transmitted stress was very close to the sum of the incident and reflected stress, which can prove that the specimen was in dynamic equilibrium throughout the SHPB test and the experimental data processing was effective [46].

#### 3.2.2. Damage and Fracture of the Specimens

**Figure 6:**
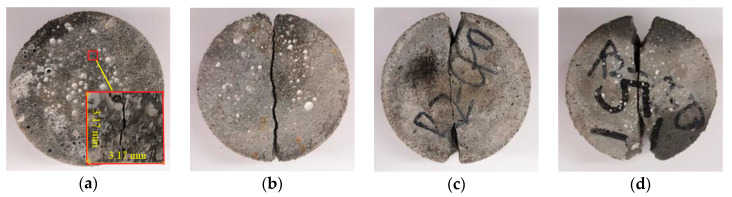
Failure patterns of specimens under room temperature and various strain rates (the incident bar is above the specimen), (**a**) 14.86 s^−1^, (**b**) 18.84 s^−1^, (**c**) 23.84 s^−1^, (**d**) 26.32 s^−1^.

The failure patterns of specimens after high temperatures were similar to those at room temperature with the change of strain rate and only the degree of damage increased with the elevation of temperature. The failure patterns of the specimens at room temperature are shown in Figure 6.

Under the low strain rate of 14.86 s^−1^, the specimen generated a macroscopic main crack along the loading direction of the incident bar and the transmission bar. When the strain rate increased to 18.84 s^−1^, secondary micro-cracks and small triangular fracture zones appeared at both ends of the specimen along the main crack, and the specimen was split into two parts. As the strain rate continued to increase, the area of the triangular fracture zone at both ends of the specimen increased gradually, and the overall deformation of the specimen became larger.

The main reason was that the damage evolution occurred first in the micro-defects near the center of the specimen due to the impact load in the splitting tensile test and rapidly expanded to the loading end face along the loading direction with a strong directionality, and finally formed a macroscopic radial main crack [47]. Before the main radial crack was connected, the compressive stress generated at the loading end face of the specimen and the compression rod increased rapidly, which caused the micro-crack initiation at the loading end face of the specimen. The generation and development of cracks were limited within the loading diameter, and so the damage degree of loading end increased and occurred as a local crushing phenomenon, which finally formed a triangular failure zone.

**Figure 7:**
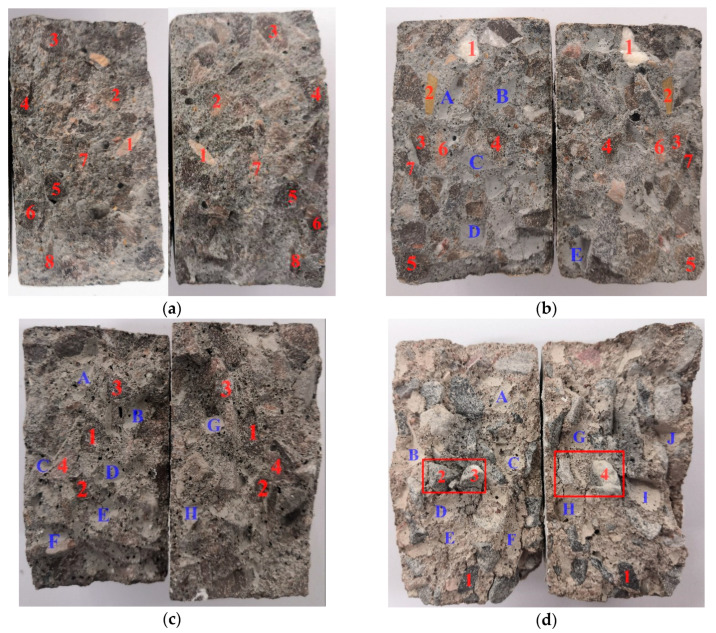
Fracture morphology under different temperatures with 0.20 MPa impact pressure (aggregate particles marked by the same number on fracture surfaces of the left and right halves are fragments of the same initial particle), (**a**) 25 °C, (**b**) 200 °C, (**c**) 400 °C, (**d**) 600 °C.

Figure 7 shows the fracture morphology of two halves under different temperatures with 0.20 MPa impact pressure.

From Figure 7a, there was the breakage of many coarse aggregate particles on the fracture surface at room temperature, and several of them, i.e., particles 1, 2, …, 8, are marked. Therefore, in these splitting tests at room temperature, when the crack propagated in concrete and met coarse aggregate particles, it did not change its propagation direction in the mortar matrix but went right through the aggregate particles, which led to the fracture of the aggregate particles. As can be seen in Figure 7b, in addition to some broken coarse aggregate particles that appeared on the fracture surface of the specimen after 200 °C, the interfaces between the coarse aggregate and mortar after debonding were also accompanied, and several of them—interfaces A, B, C, and D—are marked.

From Figure 7c,d, the number of interfaces between the coarse aggregate and mortar on the fracture surface of the specimen increased after the 400 °C high temperature. After the 600 °C high temperature, the coarse aggregate and mortar matrix were almost completely debonded, and a large number of coarse aggregate particles remained intact and cracks occurred between them and the mortar matrix as can be seen at the numbers 2, 3, and 4 in Figure 7d.

#### 3.2.3. The Dynamic Stress–Strain Relationship of the Concrete after High Temperature 

**Figure 8:**
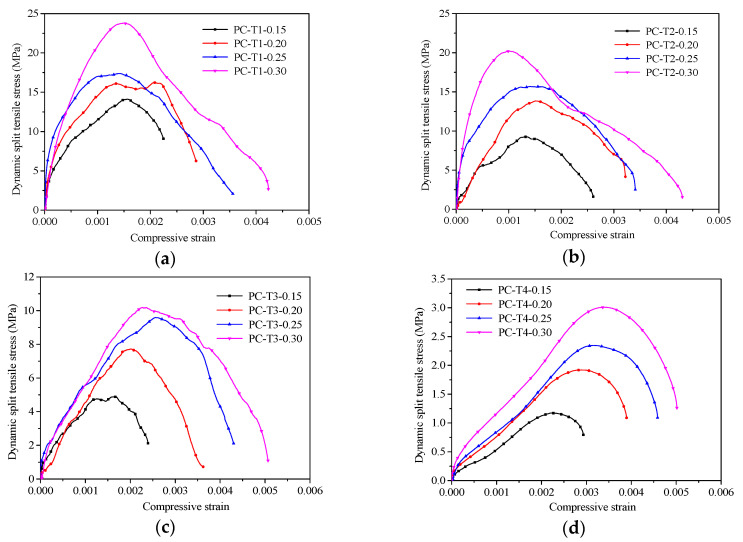
The stress-strain curves of specimens under various impact pressures and temperatures, (**a**) 25 °C, (**b**) 200 °C, (**c**) 400 °C, (**d**) 600 °C.

To demonstrate the change in the dynamic tensile strength after high temperature more clearly, the experimental pulse waveforms were calculated and processed according to Equations (2)–(4). Then, the stress–strain curves of specimens under various impact pressures and temperatures were obtained as shown in Figure 8.

As shown in Figure 8, generally, the four different temperature treatments of the dynamic tensile stress along with the change of compressive strain had a similar rule. Compared with the static test, the dynamic tensile strength of concrete under different temperatures all increased with the rise of the strain rate. The shape of the stress–strain curves was unchanged, and the corresponding dynamic strength was significantly greater than the corresponding static value, which showed the significant effect of the strain rate. This was because the failure of the concrete specimens was caused by the generation and development of cracks. 

In general, the energy needed for the crack formation was bigger than that for the crack development, the destruction of the concrete test specimen often propagated along the interface of the aggregate and mortar with the lowest strength [48]. In the dynamic test, the impact duration was short, the specimen did not have enough time to accumulate energy, the fracture occurred before the crack was fully extended along with the weak interface, or the fracture was not necessarily spread along the interface of the aggregate and mortar. 

After the test, we also found that the coarse aggregate at the section was sometimes split directly and the fracture surface was smooth (Figure 7a). This phenomenon made the tensile strength of the concrete increase with the increase in the strain rate. It was also clear that the dynamic tension strength of the concrete decreased with the increase in temperature, and the stress–strain curves tended to be flat. The strength of high-strength concrete demonstrated a temperature softening effect and a strain rate strengthening effect.

#### 3.2.4. The Dynamic Tension Strength of Concrete after High Temperatures 

**Figure 9:**
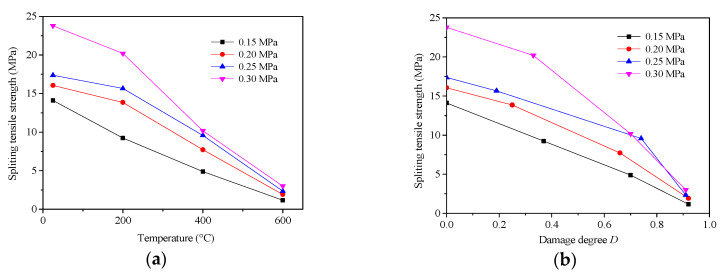
The relationship between (**a**) the splitting tensile strength and temperature; and (**b**) the dynamic splitting tensile strength and damage degree.

Figure 9a exhibits the relationships between the splitting tensile strength and temperature under different impact pressures in the experimental range.

Figure 9b shows the relationships between the dynamic splitting tensile strength and damage degree at different impact pressures. Figure 9a,b combined show that, under the same impact pressure, the dynamic tension strength of concrete under high temperature reduced significantly with the rising of temperature, and particularly after temperatures over 400 °C, the tensile strength fell sharply. When the temperature reached 600 °C, the damage degree reached more than 90%, and this changing trend shows that the high temperature dynamic tensile strength of concrete had an obvious damage degradation effect, and with the increase in temperature, the damage and degradation effect on the strength increased. This can be explained by the changes in the fracture surface of the specimen shown in Figure 7.

With the increase in temperature, the fracture surface of the specimen changed from a section dominated by broken coarse aggregate to one dominated by the debonding interface between coarse aggregate and mortar. The energy required for the crack to run through the coarse aggregate was greater than that required for the expansion along with the interface between coarse aggregate and mortar. Therefore, the dynamic tension strength of the specimen decreased with the increase in temperature.

**Figure 10:**
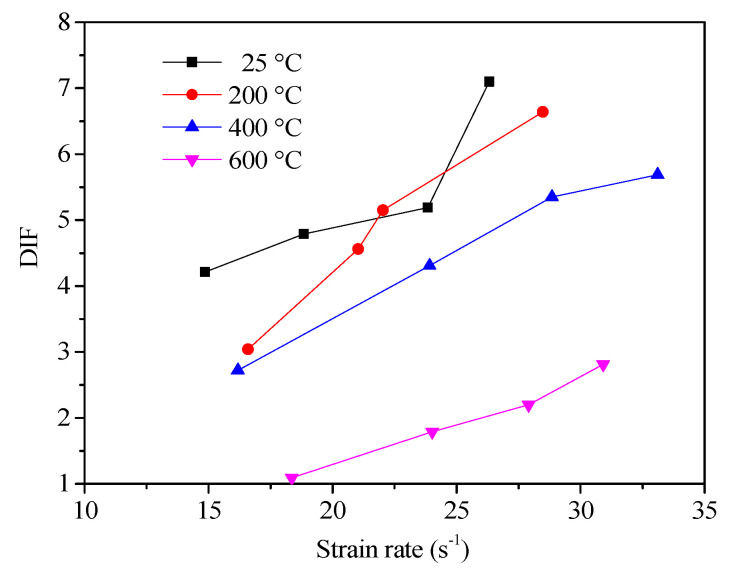
The dynamic increase factor vs. the strain rate.

Figure 10 plots the variation of DIF with the strain rate at different temperatures.

As expected, the DIF tended to increase with the rise of the strain rate, which was also observed in splitting tensile tests on concrete by other researchers [49,50]. However, the specimen had a large difference variation range of DIF with the strain rate at different temperatures. Within the experimental strain rate (14–35 s^−1^), the variation range of DIF values at room temperature, 200, 400, and 600 were 4.21—7.10, 3.04—6.64, 2.72—5.69, and 1.09—2.81, respectively. The dynamic strengthening effect of the concrete splitting tensile strength was the largest at room temperature, and the dynamic strengthening effect of concrete splitting tensile strength decreased gradually with the rise of temperature.

**Figure 11:**
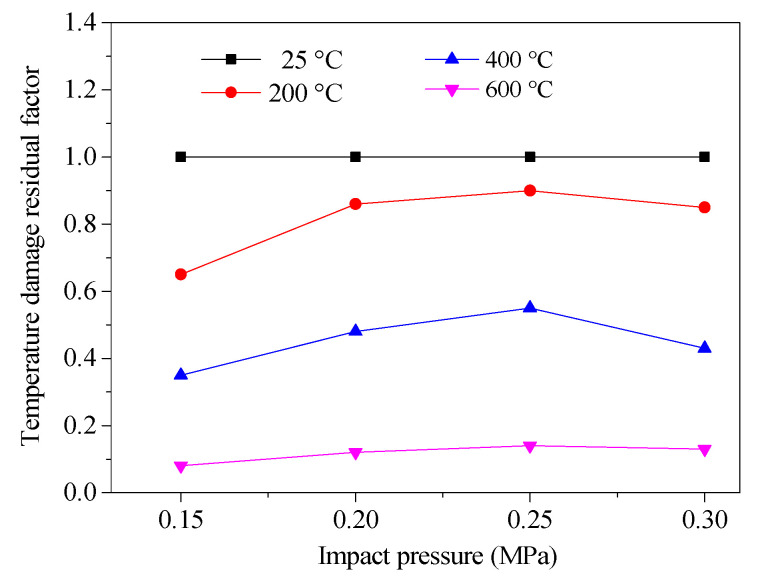
The temperature damage residual factor vs. the impact pressure.

The temperature damage residual factor (the ratio of the tensile strength after high temperatures to the tensile strength at room temperature) was used to reflect the degradation degree of the dynamic properties of concrete treated after different temperatures. Figure 11 compares the change of the temperature damage residual factor with the impact pressure of specimens treated at different temperatures.

As shown in Figure 11, under each impact pressure, the temperature damage residual factors of specimens treated after distinct high temperatures were all smaller than those at room temperature, and the temperature damage residual factors showed a decreasing trend with the rise of temperature. Simultaneously, the variation range of the temperature damage residual factor with the increase in impact pressure was very small at the same temperature, and the variation range of the temperature damage residual factor after 600 °C was only 0.05, indicating that the strain rate had no significant effect on the temperature degradation law of dynamic tensile strength.

#### 3.2.5. Energy Dissipation of Concrete after High Temperature 

In the process of impact failure, the concrete material was always accompanied by energy conversion and transfer; therefore, the failure mechanism of the concrete material can be more accurately understood from the perspective of energy dissipation. In the SHPB test, from the initial loading to the final unloading, the incident energy *W_I_*, transmitted energy *W_T_*, and reflected energy *W_R_* received by the specimen can be calculated according to the Equations (7)–(9) [48]:

(7) $$W_{I}(t)=AEC_{0}\int_{0}^{t}\varepsilon_{I}{}^{2}(t)dt$$

(8) $$W_{R}(t)=AEC_{0}\int_{0}^{t}\varepsilon_{R}{}^{2}(t)dt$$

(9) $$W_{T}(t)=AEC_{0}\int_{0}^{t}\varepsilon_{T}{}^{2}(t)dt$$

where *A* is the cross-sectional area of the bar; *E* is the elastic modulus of the bar material; *C*_0_ is the longitudinal wave velocity of the bar; and *ε_I_*(*t*), *ε_R_*(*t*), and *ε_T_*(*t*) are the incident strain, reflected strain, and transmitted strain of a certain time t in the pressure bar, respectively.

Ignoring the energy consumed by the friction between the contact surface of the incident bar and the specimen and the specimen and the transmission bar in the loading process, the energy *W_S_* absorbed by the failure of the specimen under impact action can be calculated using Equation (10).

(10) $$W_{S}(t)=W_{I}(t)-[W_{R}(t)+W_{T}(t)]$$

In the SHPB test, due to the size difference of the specimens, the data processing results were highly discrete. To minimize the errors caused by the size difference of the specimens, the specific energy absorption value, namely the crushing energy absorption of the specimen per unit volume, is introduced, Equation (11):

(11) $$\xi =\frac{W_{S}}{V_{S}}$$

where *ξ* is the specific energy absorption value of the concrete specimen; *W_S_* is the absorbed energy of the specimen; and *V_S_* is the volume of the specimen.

**Figure 12:**
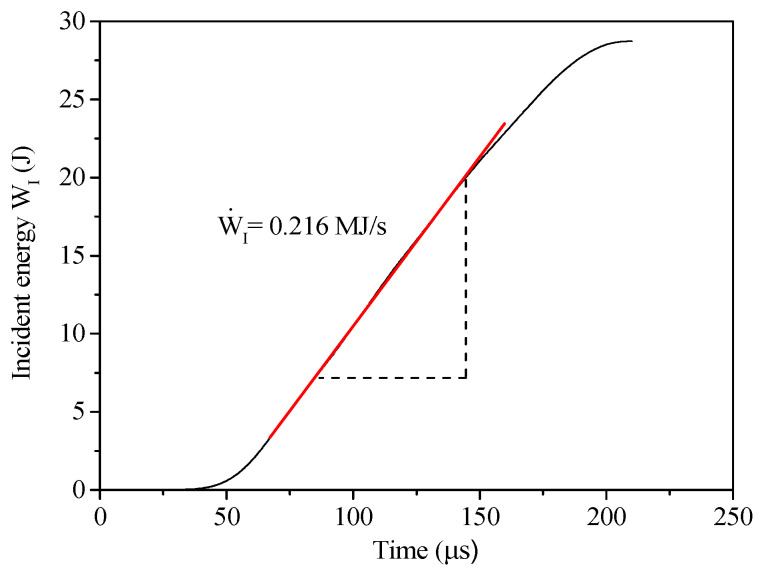
Calculated method of the average change rate of the incident energy.

To eliminate the influence of the nominal strain rate under different impact pressures on the specimen splitting strength, the average rate of change of the incident energy $\overset{•}{W_{I}}$ was defined, and the calculation method is shown in Figure 12. $\overset{•}{W_{I}}$ was completely independent of the splitting tensile strength and had a more intuitive reaction loading rate effect.

According to Equations (7)–(11), the test data were processed to obtain the energy results of the dynamic splitting test of concrete specimens treated after different temperatures, and the energy results are listed in Table 4.

**Figure 13:**
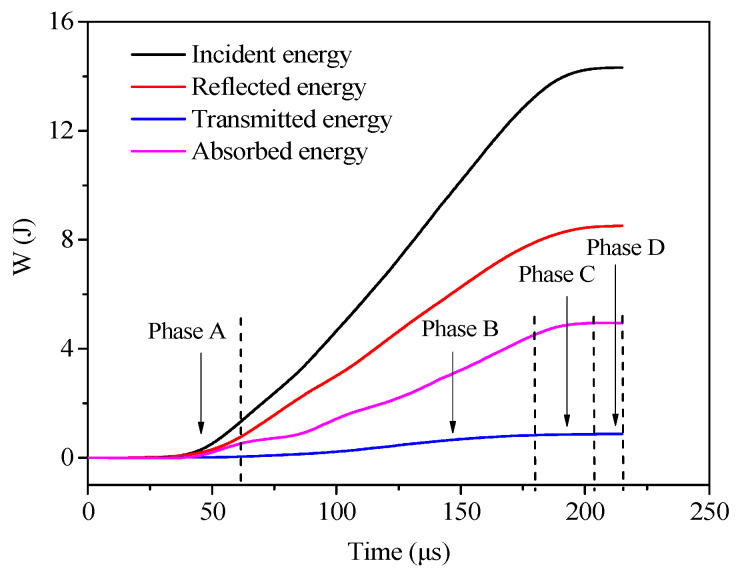
Typical energy curves of specimens.

The typical energy time–history curves of the specimens are shown in Figure 13.

The reflected energy of the specimen was large, while the transmitted energy was small and far less than the reflected energy. After passing through the specimen, only a small part of the energy was transferred to the transmission rod to form transmission energy, while a large part of the energy was absorbed by the specimen to form the absorption energy when the stress wave penetrated the specimen. From the perspective of energy absorption, the failure of the energy absorption process of the concrete specimen was divided into four stages:

1. Compaction stage (Phase A): At this time, the stress wave was on the rising edge. Under the action of the impact load, the pores in the concrete specimen were compacted and were in the compression deformation stage. The absorbed energy in the process was small, and the energy consumption curve grew slowly.
2. Elastic phase (Phase B): In this phase, the incident energy, reflected energy, and absorbed energy curves all grew rapidly. Damage evolution and accumulation occurred inside the specimen, and the specimen absorbed a large amount of energy for crack initiation and internal crack growth [51].
3. Yield stage (Phase C): In this stage, the cracks initiating inside the specimen further expanded and penetrated, and the rate of energy absorption gradually decreased, but the absorption energy continued to increase.
4. Softening stage (Phase D): At this time, the incident loading basically ended, the absorption energy did not increase, and finally, each energy tended to a stable value and the energy curve remained level, indicating that the specimen had been completely destroyed.

**Figure 14:**
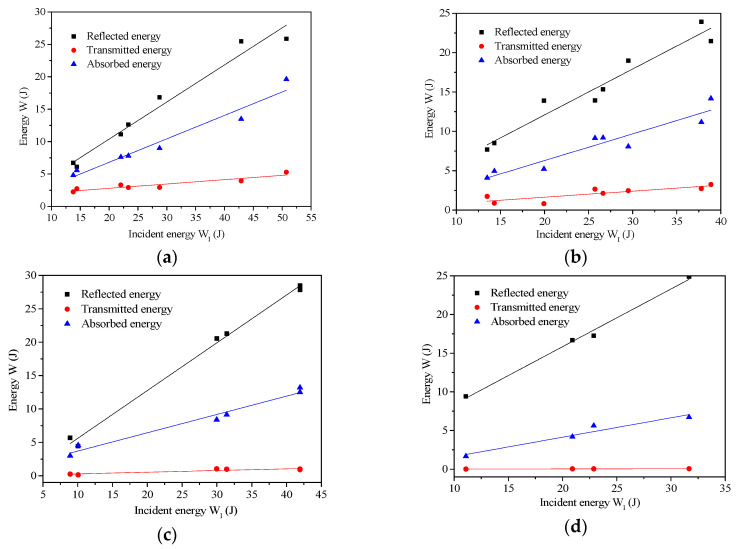
The reflection, transmission, and absorption energy vs. the incident energy, (**a**) 25 °C, (**b**) 200 °C, (**c**) 400 °C, (**d**) 600 °C.

In the SHPB splitting tensile test of the specimen, the reflection energy, transmission energy, and absorption energy of the specimen after different temperatures changed with the incident energy as shown in Figure 14.

Both the reflected energy and the absorbed energy increased with the increase in incident energy, which had a good linear relationship and the increase rate of the reflected energy was greater than the increase rate of the absorbed energy of the specimen. The transmission energy was generally stable at the same temperature, and the fitting curves were approximately a horizontal line, indicating that the transmission energy was not significantly affected by the incident energy, which may be related to the deformation and failure of the specimen.

**Figure 15:**
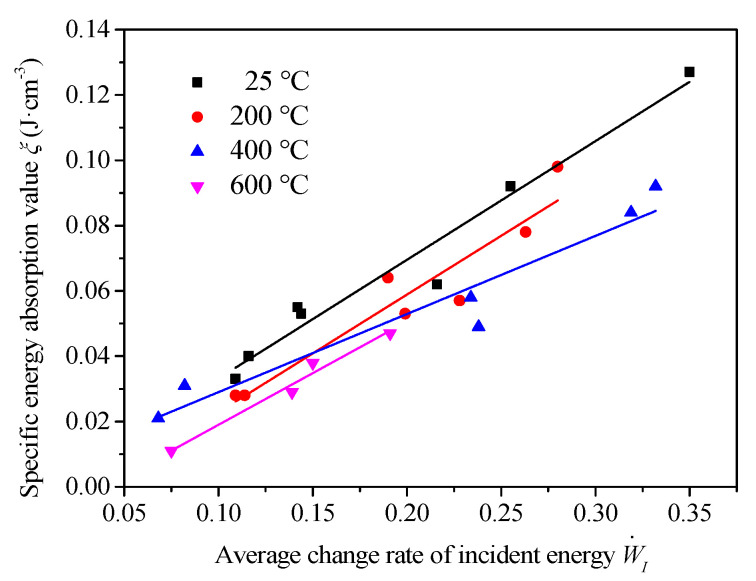
The specific energy absorption vs. the average change rate of incident energy.

The relationship between the specific energy absorption value and the average change rate of incident energy treated after different temperatures is shown in Figure 15.

The specific energy absorption value of the specimen after distinct temperatures increased with the rise of the average rate of change of incident energy, and had a good linear relationship as described in Table 5.

At the same time, as the temperature increased, the specific energy absorption value showed a downward trend. With the increase in temperature, the fracture surface of the specimen changed from a section dominated by broken coarse aggregate to one dominated by the debonding interface between the coarse aggregate and mortar, and the energy required for the crack to run through the coarse aggregate was greater than that required for the expansion along with the interface between coarse aggregate and mortar. Therefore, the absorption energy of the specimen decreased with the rise of temperature. Because the size of the sample did not change, the specific energy absorption value also decreased with the increasing temperature.

## 4. Conclusions

This work used the SHPB test system to conduct dynamic splitting tensile tests on high-strength concrete treated with different temperatures. The dynamic splitting tensile strength and dynamic enhancement coefficient of high-strength concrete after high temperatures were obtained, the damage and fracture characteristics of high strength concrete after different temperature treatments were observed, and the energy change characteristics of high-strength concrete were analyzed. The following conclusions were drawn:

1. With the increase in temperature, the quasi-static and dynamic tensile strength of high strength concrete gradually decreased, and the damage degree of the specimen gradually increased.
2. The dynamic tensile strength and specific energy absorption of high-strength concrete increased with the increase in the strain rate, but decreased with the increase in temperature, indicating that high-strength concrete had both a strain rate effect and temperature softening effect.
3. In the dynamic splitting tensile test, when the high-strength concrete at room temperature was subjected to an impact load, the direction of the crack propagation in the mortar matrix did not change, but directly penetrated the coarse aggregate, causing the coarse aggregate particles to break, which was a brittle failure. With the increase in temperature, the debonding surface of the coarse aggregate and mortar matrix increased, and the number of broken coarse aggregates decreased, indicating that the crack propagation law gradually changed from directly passing through the coarse aggregate to extending along the bonding surface between the coarse aggregate and the mortar matrix.
4. Based on the above test results, temperature had a great influence on the dynamic tensile properties of high-strength concrete. Based on the failure patterns of the specimens at high temperature, when high-strength concrete structures will be under environments of high temperatures and dynamic loads, the bond between the mortar and aggregate should be strengthened in the material proportioning design. Materials with high-temperature resistance and good tensile strength can be added to change the failure mode of the structure. The experimental results have reference significance for guiding the fire response and seismic design of high-strength concrete.

## Figures and Tables

**Table 1:** The ratio of concrete materials (kg/m^3^).

Cement	Water	Fine Aggregate	Coarse Aggregate	Coal Ash	Silica Fume	Water Reducing Agent	Rubber
385.9	154.36	699	1140	45.4	22.7	7.8	9.08

**Table 2:** The chemical composition and geometric characteristics of cement and silica fume.

Component	Density (kg/m^3^)	BET (m^2^/g)	Chemical Composition (wt.%) (XRF)					
			CaO	SiO_2_	Al_2_O_3_	Fe_2_O_3_	SO_3_	MgO
Cement	1910	1.477	31.31	1.94	0.9	0.23	43.49	0.29
Silica fume	310	23.7	0.8	97	0.6	0.1	1.0	-

**Table 3:** The mechanical properties of concrete under dynamic splitting tensile tests.

Temperature (°C)	Gas Pressure (MPa)	Wave Velocity (m/s)		Strain Rate (s^−1^)	Damage Degree *D*	Dynamic Tensile Strength (MPa)	DIF
		*c*_1_	*c*_2_				
25	0.15	5240	5240	14.86	0	14.11	4.21
	0.20	5102	5102	18.84	0	16.06	4.79
	0.25	5454	5454	23.84	0	17.38	5.19
	0.30	5305	5305	26.32	0	23.79	7.10
200	0.15	5438	4306	16.59	0.37	9.24	3.04
	0.20	5329	4602	21.03	0.25	13.85	4.56
	0.25	5185	4667	22.03	0.19	15.67	5.15
	0.30	5213	4265	28.48	0.33	20.20	6.64
400	0.15	5153	2810	16.18	0.70	4.88	2.72
	0.20	5000	2903	23.92	0.66	7.72	4.31
	0.25	5379	2759	28.85	0.74	9.58	5.35
	0.30	5153	2811	33.11	0.70	10.18	5.69
600	0.15	5231	1519	18.36	0.92	1.17	1.09
	0.20	5013	1380	24.02	0.92	1.92	1.79
	0.25	5167	1583	27.91	0.91	2.35	2.20
	0.30	5106	1507	30.91	0.91	3.01	2.81

**Table 4:** Energy results of the SHPB test of concrete at different temperatures.

Specimen Number	Temperature (°C)	*W_I_* (J)	*W_R_* (J)	*W_T_* (J)	*W_S_* (J)	${\overset{•}{W}}_{I}\;(\mathrm{MJ}/s)$	*ξ* (J/cm^3^)
PC-T1-1	25	13.78	6.72	2.24	4.82	0.109	0.033
PC-T1-2		14.40	6.11	2.71	5.58	0.116	0.040
PC-T1-3		23.34	12.62	2.91	7.81	0.142	0.055
PC-T1-4		22.03	11.12	3.30	7.61	0.144	0.053
PC-T1-5		28.74	16.82	2.94	8.98	0.216	0.062
PC-T1-6		42.89	25.47	3.94	13.48	0.255	0.092
PC-T1-7		50.73	25.85	5.27	19.61	0.350	0.127
PC-T2-1	200	14.31	8.50	0.87	4.94	0.114	0.028
PC-T2-2		13.50	7.68	1.73	4.09	0.109	0.028
PC-T2-3		26.64	15.34	2.12	9.18	0.199	0.053
PC-T2-4		29.51	18.97	2.46	8.08	0.228	0.057
PC-T2-5		25.73	13.93	2.65	9.15	0.190	0.064
PC-T2-6		38.88	21.46	3.25	14.17	0.280	0.098
PC-T2-7		37.80	23.92	2.72	11.16	0.263	0.078
PC-T3-1	400	8.91	5.67	0.25	3.00	0.068	0.021
PC-T3-2		10.07	4.44	0.14	4.58	0.082	0.031
PC-T3-3		31.41	21.28	0.99	9.14	0.238	0.049
PC-T3-4		29.98	20.54	1.04	8.39	0.234	0.058
PC-T3-5		41.94	28.45	1.00	12.49	0.319	0.084
PC-T3-6		41.94	27.84	0.91	13.19	0.332	0.092
PC-T4-1	600	11.09	9.42	0.01	1.66	0.075	0.011
PC-T4-2		20.91	16.68	0.04	4.19	0.139	0.029
PC-T4-3		22.88	17.24	0.04	5.61	0.150	0.038
PC-T4-4		31.67	24.90	0.05	6.71	0.191	0.047

**Table 5:** Energy results of the SHPB test of concrete after different temperatures.

Temperature (°C)	Equation	Correlation Coefficient (R^2^)
25	$\xi =0.3635{\overset{•}{W}}_{I}-0.0032$	0.9511
200	$\xi =0.3603{\overset{•}{W}}_{I}-0.0132$	0.8843
400	$\xi =0.2393{\overset{•}{W}}_{I}+0.0051$	0.9116
600	$\xi =0.3163{\overset{•}{W}}_{I}-0.0126$	0.9657
